# Do Fibroblast Growth Factor Receptor (FGFR) 2 and 3 Proteins Play a Role in Prognosis of Invasive Urothelial Bladder Carcinoma?

**DOI:** 10.30699/IJP.2024.2012115.3180

**Published:** 2024-03-29

**Authors:** Shereen Fathy Mahmoud, Nanis Shawky Holah, Alshimaa Mahmoud Alhanafy, Marwa Mohammed Serag El-Edien

**Affiliations:** 1 Department of Pathology, Faculty of Medicine, Menoufia University, Minufiyah, Egypt; 2 Department of Clinical Oncology and Nuclear Medicine, Faculty of Medicine, Menoufia University, Minufiyah, Egypt

**Keywords:** FGFR2, FGFR3, Immunostaining, Prognosis, Urothelial carcinoma

## Abstract

**Background & Objective:**

Bladder carcinoma ranks second in prevalence among males in Egypt. As a family of tyrosine kinases, fibroblast growth factor receptor (FGFR) dysregulation has been linked to some malignancies in humans. The aim of this study is to analyze the clinicopathological data of patients while investigating FGFR2 and FGFR3 immunohistochemical expression in invasive urothelial bladder carcinoma.

**Methods:**

This retrospective cross-sectional study included 60 invasive urothelial carcinoma (UC) cases in the Pathology department, Faculty of Medicine, Menoufia University, from 2009 to 2020. All biopsies were stained for FGFR2 and FGFR3 antibodies. Complete clinical data were available for 44 patients treated and followed in clinical oncology and nuclear medicine departments.

**Results:**

Advanced stage and high grade are significantly correlated with FGFR2 positivity (*P*=0.048 and 0.044, respectively). Cases presented with Perineural invasion showed a higher percentage of FGFR2 (*P*=0.023). There is a significant indirect linear correlation between FGFR3 expression and lymph node positivity (r= -0.265, *P*=0.041).

**Conclusion:**

A high FGFR2 expression could be associated with poor prognostic parameters, while high FGFR3 expression would be associated with good prognostic parameters. These findings might highlight the importance of FGFR-targeted therapy as a FGFR2 antagonist and FGFR3 agonist for the treatment of urothelial carcinoma patients.

## Introduction

Bladder carcinoma is one of the most prevalent malignant tumors involving the urinary system. It ranks tenth in global cancer incidence (1). In 2023, it has been considered as the fourth most common cancer in men in the United States after prostate, lung, and colon cancers. Bladder carcinoma comprises 6% of the projected new cases of cancer, with an estimated mortality rate of 12.160 (2). 

Bladder cancer ranks as the third most prevalent malignant tumor in Egypt (excluding melanoma skin cancer), with 7.9% as estimated new cancer cases in both sexes. It is the 2^nd ^most prevalent cancer among men, by the exclusion of melanoma skin cancer after liver cancer. Bladder carcinoma is responsible for an estimated 12.6 % of newly diagnosed cancer cases. Moreover, it occupies the third place in cancer death causes after liver and breast cancer, accounting for 6.9% of deaths (3).

The fibroblast growth factor receptors (FGFRs) comprise four distinct receptors and are a family of tyrosine kinases: FGFR1 and FGFR4 (4). Although these receptors are encoded by distinct genes, they all possess a high degree of sequencing identity (5). These receptors are situated on the cell membrane and comprise intracellular, transmembranous, domains, and extracellular. The variation among FGFRs is primarily attributable to alternative splicing of the mRNA sequence responsible for generating the extra-membranous domain (6). FGFR2 is implicated in cell migration, angiogenesis, survival, and proliferation, among other cellular processes. It is consistent with its expression in numerous tissues and is vulnerable to dysregulation in cancer cells. The context-dependent activation of the FGFR2 pathway can induce both oncogenic and tumor-suppressive effects (7).

Urothelial carcinoma (UC) is among the human malignancies to which dysregulation of FGFRs has been linked. The RAS-mitogen-activated protein kinase pathway mediates the oncogenic function of mutated FGFR in UC, comparable to the effects mediated by activated HRAS (8).

FGFR3 mutations and fusions are observed in 10–20% of metastatic UCs; these tumors are susceptible to FGFR inhibitors. However, acquired resistance to these agents may develop, resulting in quite often short-term life (9).

The Food and Drug Administration (FDA) granted approval in April 2020 for erdafitinib, the initial targeted FGFR therapy, specifically indicated for patients diagnosed with metastatic bladder or locally advanced cancer who had not responded favorably to platinum-based chemotherapy (10).

Urine cytology and cystoscopy are frequent diagnostic techniques for bladder carcinoma (BC). Nevertheless, standard white light cystoscopy, which neglects the presence of narrow-band imaging and fluorescence, may fail to detect small papillary tumors. Hence, in light of the varying incidence ratios across countries and regions, it is necessary to develop population-based innovative diagnostic and screening methodologies to promptly identify high-risk cases and BC (11). 

Also, because of the importance of FGFR, since FGFR genomic alterations have become a cornerstone in urinary carcinoma (UC), Our objective was to determine whether immunohistochemical expression of FGFR2 and FGFR3 in UC patients is prognostic and to establish a correlation between their expression and the clinicopathological parameters that are currently available.

## Material and Methods

**Study Cohort and Pathological Evaluation:**

This retrospective cross-sectional study involved 60 cases of invasive urothelial carcinoma (IUC). The cases were carried out in the Pathology Department, Faculty of Medicine, Menoufia University from 2009 to 2020. The included cases (60 cases) were IUC type, either pure or with squamous differentiation, and did not receive neoadjuvant therapy before obtaining the specimen. Exclusion criteria from survival analysis were considered as follows: patients with incomplete data or unavailable files from the studied cohort. Forty-four patients were known to be eligible with complete clinical data. All were treated and followed in the Clinical Oncology and Nuclear Medicine Department, Faculty of Medicine, Menoufia University.

Histopathological and clinical information regarding cases was extracted from patients' pathology records. Age (<60 years old) was included in the data (12), grade, gender, T stage (Assigned the "at least T2 stage" for IUC cases resected via transurethral resection of bladder tumor), N stage in cystectomy specimens (The cases were categorized as having negative nodal status [N0] or positive nodal status [N1, 2 & 3].) (13). The assessment included the evaluation of lympho-vascular invasion (LVI), bilharziasis, perineural invasion (PNI), and necrosis.

Other clinical and treatment outcome Data were collected from the paper files, including family history, smoking, disease TNM Stage at presentation, performance status, presence of anemia and/or hydronephrosis at presentation, presence of comorbidities, treatment received survival, and metastatic disease site. Staging according to the AJCC cancer TNM staging manual eighth edition 2017 (14), assessment of performance status was done using the Eastern Cooperative Oncology group scale (15). Progression-free survival (PFS) was computed from the date of diagnosis to progression. The date of diagnosis to death or last contact was utilized to compute overall survival (OS).

**Immunohistochemical (IHC) Staining**

Tissue microarray blocks were prepared from the cystectomy specimens. Four-μm-thickness sections were cut from each tissue block. The 1ry antibodies used were polyclonal rabbit FGFR2 (isotype IgG, 0.1ml concentrated, Chongqing Biopsies Co., Ltd, Chongqing, China, Catalog No.:YPA 2200) and monoclonal rabbit FGFR3 (isotype IgG, 0.1ml concentrated, AB clonal, Woburn, United States, Catalog No.: A19052). Both antibodies are used at dilution 1:100. Human tonsil and esophageal cancer tissues were used as positive control for FGFR2 & 3, respectively. The primary antibody step was replaced with a blocking buffer in the IHC procedure as a negative control.

**Interpretation of the Immunostaining Results**

FGFR2 and FGFR3 positivity was indicated on the tumor cell membrane by brown staining. Both markers were assessed utilizing the subsequent methodologies: Expression (negative/positive): A positive result was obtained when the cell membranes of any number of malignant cells exhibited brown staining.

The percentage of positivity (%) was assessed using measures such as the median, mean, and range. Predominant intensity of staining: evaluated as negative, mild, moderate, and strong. For statistical purposes, negative and mild were grouped as low, moderate, and strong as high intensity.

The immunoreactivity score (IRS) is determined by the percentage of the intensity and positivity of the staining, as stated by Kindler* et al.* (2004) (16). Zero to 12 was its range, which was calculated using the median, mean, and range.

**Statistical Analysis**

Data were statistically analyzed using Statistical Package of Social Science (SPSS) version 23 (SPSS, Inc., Chicago, Illinois, USA). 

The range, standard deviation (SD), and mean are utilized to represent quantitative data. Numbers and percentages are used to represent qualitative data. The Chi-square test (χ2) was used to examine the association between two qualitative variables. Mann Whitney and Kruskal tests to compare between non-normally distributed two and more than two quantitative variables, respectively. Spearman coefficient correlates between two non-normally distributed quantitative variables. 'Time-to-event' data analysis was analyzed using Kaplan-Meier. Cox regression is utilized for measuring the effect of several variables upon the time a specified event takes to happen. A confidence interval (CI) of 95% was utilized. It is deemed statistically significant to have a P-value<0.05 and highly significant to have a P-value<0.001.

## Results

**Clinicopathological Data of the Studied Infiltrating UC Cases**

Table 1 presents the pathologic and clinical data of the examined cases.

**Table 1:** Clinicopathological data of the studied IUC cases (n = 60)

	**Variable**		**No. (%)**
	**Age (years)**	Mean ± SD	61.45 ± 8.2
		Median	63
		Min. – Max.	23 - 80
	**Gender**	Male	51 (85)
		Female	9 (15)
	**Specimen**	TURT	15 (25)
		Cystectomy	45 (75)
	**Histological type**	Urothelial	33 (55)
		Urothelial with squamous differentiation	27 (45)
	**Grade**	Low	3 (5)
		High	57 (95)
	**T stage**	T2	15 (25)
		T3	22 (36.7)
		T4	8 (13.3)
		at least T2	15 (25)
		at least T2/T2	30 (50)
		T3	22 (36.7)
		T4	8 (13.3)
**In cystectomy cases (n=45)**	**N stage **	NA	2 (4.4)
		N0	27 (60)
		N1	5 (11.2)
		N2	11 (24.4)
	**No. of Pos. LNs**	Mean ± SD	4.75 ± 5.2
		Median	2.5
		Min. – Max.	1 - 21
	**N stage groups**	NA	2 (4.4)
		Negative	27 (60)
		Positive	16 (35.6)
	**Bilharziasis**	Absent	40 (66.7)
		Present	20 (33.3)
	**Necrosis**	Absent	41 (68.3)
		Present	19 (31.7)
	** PNI**	Absent	41 (70)
		Present	18 (30)
	** LVI**	Absent	46 (76.7)
		Present	14 (23.3)

**Fig. 1.**
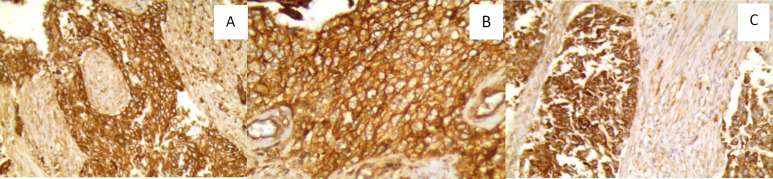
(A): Muscle invasive urothelial bladder carcinoma (UBC) positive for FGFR2, showing perineural invasion (IHCx200). (B): High grade UBC showing FGFR2 positivity (IHCx400). (C): Muscle invasive UBC showing FGFR3 positivity (IHCx200)

**Immunostaining of FGFR2 &FGFR3 (** Figure 1 **)**

Table 2 contains the outcomes of FGFR2 and 3 immunostaining in terms of expression (positive/ negative), percentage of the positive cases, and intensity.

**Table 2:** Results of the immunostaining of FGFR2 & 3

		**IHC**	**No. (%)**
**FGFR2 **	**Expression**	Negative	8 (13.3)
		Positive	52 (86.7)
	**Intensity**	Negative	8 (13.3)
		Mild	20 (33.3)
		Moderate	28 (46.7)
		Strong	4 (6.7)
	**Intensity groups**	Low intensity	28 (46.7)
		High intensity	32 (53.3)
	**I** **RS score**	Mean ± SD	4.1 ± 2.4
		Median	4
		Min. – Max.	0 - 9
	**Percentage**	Mean ± SD	54.33 ± 26.3
		Median	60
		Min. – Max.	0 - 90
**FGFR3 **	**Expression**	Negative	7 (11.7)
		Positive	53 (88.3)
	**Intensity**	Begative	7 (11.7)
		Mild	7 (11.7)
		Moderate	34 (56.6)
		Strong	12 (20)
	**Intensity groups**	Low intensity	14 (23.3)
		High intensity	46 (76.7)
	**IRS score**	Mean ± SD	5.7 ± 3.2
		Median	6
		Min. – Max.	0 - 12
	**Percentage**	Mean ± SD	61.83 ± 27.7
		Median	70
		Min. – Max.	0 - 95

**Relation Between FGFR2 Expression and the Studied Clinicopathological Parameters**

**Fig. 2:**
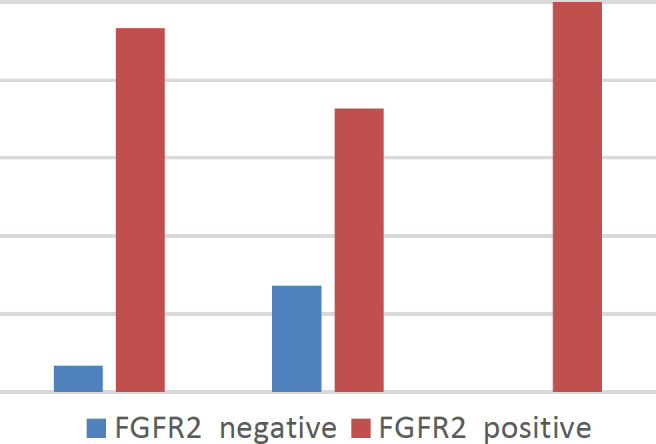
All T4 cases showing positive FGFR2 expression (*P*=0.048)

**Fig. 3:**
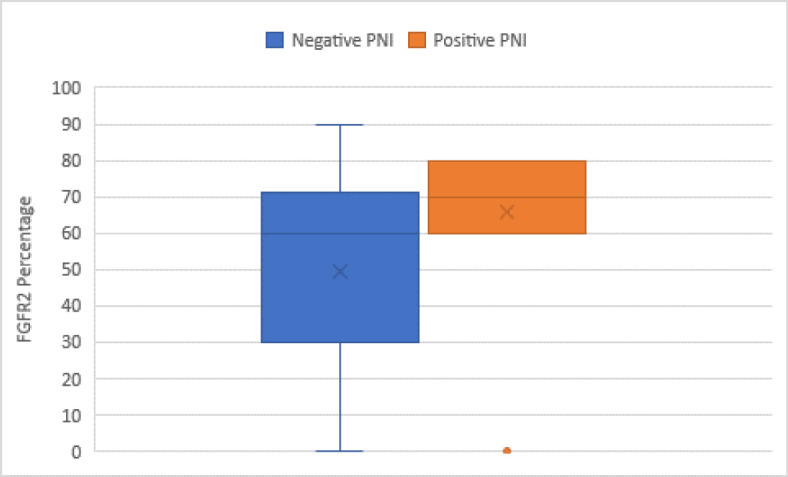
Cases with positive PNI showing high FGFR2 percentage (*P*=0.023)

High grades and advanced stages of the disease were significantly associated with FGFR2 positivity (*P*=0.044 and 0.048, respectively) (Figure 2). Also, the median FGFR2 IRS was higher in the high-grade cases (*P*=0.027). Moreover, cases presented with PNI showed a higher average percentage of FGFR2 (*P*=0.023) (Figure 3). 

**Relation Between FGFR3 Expression and the Studied Clinicopathological Parameters**

**Fig. 4:**
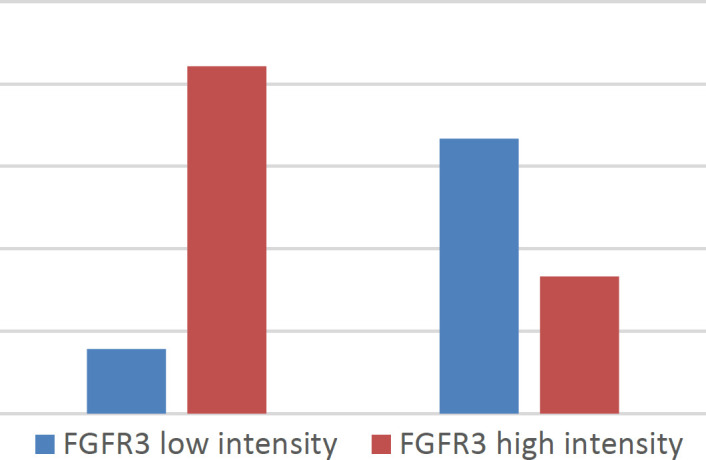
**The **male cases showing high intensity of FGFR3 (*P*=0.003)

**Fig. 5:**
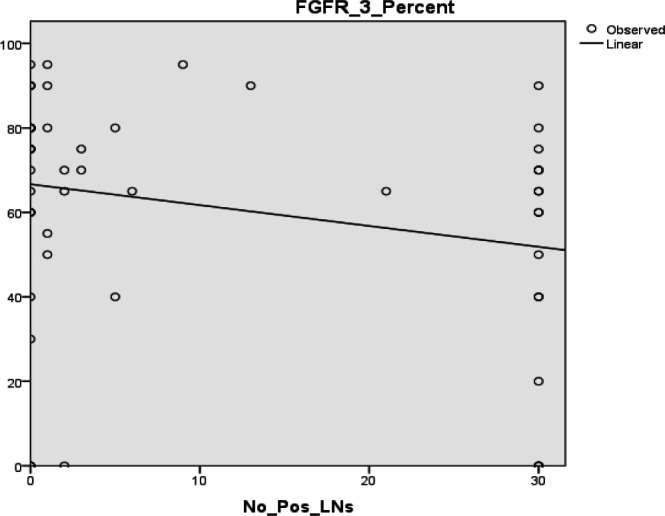
Increased number of positive lymph nodes is associated with a significant indirect linear correlation with FGFR3 percent (r= -0.265, *P*=0.041)

Male cases showed a significantly high intensity of FGFR3 (*P*=0.003) (Figure 4*). *A significant indirect linear correlation was found between FGFR3 percent of expression and the number of positive lymph nodes (r= -0.265, *P*=0.041) (Figure 5*).*

**Correlation Between FGFR2 &FGFR3:**

There was no statistically significant correlation between both markers.

**Survival Analysis**

For 44 cases of muscle-invasive UBC in the cohort under study, complete medical records were accessible. The treatment, clinicopathological, demographic, and immunohistochemical data are described in (Table 3).

Regarding treatment, in this retrospective study, we found that most of the included patients (83.3%) underwent radical cystectomy; only 13.6% had stage IV, 25% had anemia at presentation and 29.5% had hydronephrosis at presentation. Radiotherapy was received in 36.4% of the patients (adjuvant/ concurrent/ palliative). Most of the patients (70.5%) received chemotherapy concurrent, palliative, adjuvant, or neoadjuvant. Five received platinum-based chemotherapy, either cisplatin or carboplatin, 6 patients received palliative chemotherapy, and 1 patient received a taxane-based regimen caused by renal impairment. 

After median follow-up duration of the cases, which was 39 months, the mean PFS and OS were 47.3 (95% CI= 38.7-56) and 47.4 (95% CI= 39.4-55.5) months, respectively. OS duration was found to be shorter in patients with advanced TNM stage, distant metastasis, and anemia at presentation, according to univariate analysis (*P*= 0.007, 0.011, and 0.002, respectively). Performance status 0 was associated with longer survival but didn't reach the statistically significant level (*P*=0.062). Regarding PFS, the univariate analysis showing the presence of hydronephrosis at presentation was associated with shorter PFS (*P*=0.029)*. *Moreover, as with the presence of anemia at presentation, OS and distant metastasis negatively affected PFS and the advanced TNM stage (*P*= 0.004, 0.035, and 0.014, respectively). Multivariate Cox regression analysis for PFS and OS revealed that there was no independent prognostic factor affecting PFS or OS. 

Regarding Figures 3 & 4 immunostaining results, no significant effect on either OS or PFS was noted. Also, other parameters such as age, sex, smoking, comorbidities, bilharziasis, other histopathological data, and treatment data were statistically insignificant as prognostic factors for OS and PFS (*P*>0.05).

**Table 3:** Distribution of the studied cases according to the different parameters (n = 44)

	**No. (%)**
**Family history of malignancy **	
No	37 (84.1%)
Yes	7 (15.9%)
**Smoking **	
Non-Smoker	16 (36.4%)
Smoker	28 (63.6%)
**Comorbidities **	
No	24 (54.5%)
Yes	20 (45.5%)
**Performance status **	
0	21 (47.7%)
1 & 2	23 (52.3%)
**Anemia at presentation**	
No	33 (75.0%)

## Discussion

As a result of varying incidence ratios between the countries and regions, it is critical to develop innovative screening and diagnostic techniques for early detection of high-risk and BC cases (11). 

Also, since FGFR genomic alterations have developed into a cornerstone in UC, our objective was to evaluate FGFR3's prognostic role in the UC cases and immunohistochemical expression of FGFR2 and to correlate their expression with the available clinicopathological parameters. 

In this study, 88.3% of the cases showed positive expression FGFR3 and these results are near that of Al-Obaidy* et al.* (2021) (17) who reported that 70% of UC showed that FGFR3 mutations and also near the results of Ikeda *et al.* (2022) (18) who demonstrated that FGFR3 immunohistochemical overexpression was observed in 90.8% of UC cases.

This study identified a significant association between high FGFR2 expression and higher median IRS and poor prognostic parameters as T4 stage, presence of PNI, and high-grade tumors. 

These results agreed with Di Martino* et al.* (2016) (19) who found the association of FGFR2 with poor prognostic parameters such as high tumor stage and grade, and enhanced ability to produce metastases, as well as an association of epithelioid phenotype with increased expression of the FGFR2 IIIc isoform in a model of bladder cancer metastasis. They suggested FGFR2 may play a role in the mesenchymal–epithelial transition, which is necessary for the later phases of development of the metastatic tumors. Thus, FGFR2 may perform distinct roles during different phases of bladder tumor progression (20). 

On the other hand, these results are not in parallel with other studies that demonstrated a possible suppressor role of FGFR2 for the bladder urothelium tumors (21); and human bladder UC that showed lower FGFR2 expression were associated with unfavorable prognosis (22 and 23). Also, Diez de Medina* et al.* (1997) (24) demonstrated an association of FGFR2 expression reduction with decreased survival.

 One possible explanation for these controversies is that decreased FGFR2 expression was associated with decreased E-cadherin expression, suggesting that loss of expression has a relation with a less adherent epithelial phenotype (25). In fact, functional studies of FGFR2-IIIb in bladder tumor cells have indicated a tumor suppressor role (26).

While erzafitinib is the initial FGFR antagonist authorized by the FDA (2019) for management of muscle-invasive UC associated with FGFR3 or FGFR2 mutations, numerous others are undergoing clinical evaluation, including pemigatinib, rogaratinib, and infigratinib (27, 28). 

The current study demonstrated that high FGFR3 expression was associated with male gender which is considered one of the good prognostic parameters according to Andreassen* et al.* (2018), demonstrated that women have a poorer favorable prognosis than men within the initial two years following diagnosis (29). Furthermore, Uhlig* et al.* (2018) provided evidence that female patients undergoing radical cystectomy for bladder carcinoma had lower overall, cancer-specific, and disease-free survival rates compared to male patients (30). 

The current research demonstrated that FGFR3 expression was associated with other good prognostic parameters as there was a significant indirect linear correlation between FGFR3 percent of expression and the number of positive lymph nodes. These findings were consistent with those of numerous other studies that examined a relationship between FGFR3 expression and both disease progression and recurrence. It was discovered that FGFR3 mutation was linked to a decreased recurrence and progression in stage Ta tumors (31). Similarly, the presence of a FGFR3 mutation is associated with a favorable prognosis in stage T1 tumors (32). Additionally, FGFR3 mutations are associated with improved survival in invasive tumors of the upper tract (33). 

These results explained that mutant FGFR3 is extremely oncogenic in rodent fibroblasts (34). These are insufficient to prompt cellular transformation in normal urothelial tissue immortalized with human telomerase.

Regarding survival analysis, univariate survival analysis and PFS were significantly associated with high-stage, distant metastasis and urinary obstruction causing hydronephrosis and these results agreed with that of Nagy* et al.* (2018) (35), Taguchi* et al.* (2013) (36) and Mitra* et al.* (2014) (37) and should be incorporated into the treatment planning process. On the other hand, these results were against that of Kucuk* et al.* (2015) (38) who provided evidence that there was no statistically significant association between PNI, tumor stage, smoking, LVI, and concurrent OS and CIS. 

There have been reports of controversial findings in studies examining the effects of FGFR2 & 3 immunostaining results on OS and PFS. Some studies such as Sevillano* et al.* (2022) (39) came in concordance with the results in the current study demonstrating that FGFR2 & 3 immunostaining results had no significant effect on either OS or PFS.

Both FGFR2 and FGFR3 play significant roles in bladder cancer, and several ongoing early clinical trials are primarily concerned with the development of FGFR-target agents.

Complete clinical data were available for 44 patients only treated and followed in the Department of Nuclear Medicine and Clinical Oncology.

## Conclusion

 Findings of the current investigation revealed the impact of FGFR2/3 expression on the prognosis of UC as high FGFR2 expression may be associated with poor prognostic parameters, while high FGFR3 expression may be associated with good prognostic parameters. These findings might highlight the significance of FGFR-targeted therapy as an FGFR2 antagonist and FGFR3 agonist for the treatment of UC patients.

## Conflict of Interest

The authors declare that they have no potential conflicts of interest to disclose.
